# Supplementary material for: Reduced efficacy of HIV-1 integrase inhibitors in patients with drug resistance mutations in reverse transcriptase
Source: Nat Commun. 2020 Dec 1;11:5922. doi: 10.1038/s41467-020-19801-x (PMC7708638; doi:10.1038/s41467-020-19801-x)
Supplement: Supplementary file 1 — Supplementary Information [file 41467_2020_19801_MOESM1_ESM.docx]

**Supplementary Information**

**Reduced efficacy of HIV-1 integrase inhibitors in patients with drug resistance mutations in reverse transcriptase**

Mark J. Siedner, Michelle A. Moorhouse, Bryony Simmons, Tulio de Oliveira, Richard Lessells, Jennifer Giandhari, Stephen A. Kemp, Benjamin Chimukangara, Godspower Akpomiemie, Celicia M. Serenata, Willem DF Venter, Andrew Hill, Ravindra K. Gupta

Supplementary Tables

**Supplementary Table 1**. Clinical and demographic variables comparing individuals included and excluded in the ADVANCE Trial pretreatment drug resistance analytic sample

**Supplementary Table 2**. Multivariable logistic regression models for virologic success in the ADVANCE Trial comparing definitions for virologic success

**Supplementary Table 3.** Virologic success in the ADVANCE Trial by the presence of the K103N mutation prior to treatment initiation

**Supplementary Table 4.** Virologic success in the ADVANCE Trial by the presence of WHO-defined pretreatment minority drug resistance as defined by a mutation frequency 2-20%

**Supplementary Table 5**. Logistic regression models for 96-week virologic success in the ADVANCE Trial stratified by participants in the efavirenz-based and dolutegravir based arms

**Supplementary Table 6**. Comparison of pre- versus on-treatment WHO-defined reverse transcriptase drug resistance mutation patterns in the entire cohort, and by treatment group

**Supplementary Table 7.** Assessment of collinearity between included covariates in our multivariable regression model of our primary outcome

**Supplementary Table 8.** Virologic success in the ADVANCE Trial by the presence of WHO-defined pretreatment drug resistance in the entire analytic dataset and restricted to sequences with an average coverage depth >1,000X

Supplementary Figures

## **Supplementary Figure 1.** Change in log_10_ viral load from the baseline enrolment visit to week 12 by treatment arm and presence or absence of World Health Organization-defined pre-treatment drug adherence mutations to reverse transcriptase.

**Supplementary Figure 2**. Kaplan-Meier curves demonstrating time to virologic suppression by treatment arm and presence or absence of World Health Organization-defined pre-treatment drug adherence mutations to reverse transcriptase.

**Supplementary Figure 3.** Distribution of read depth coverage among samples included in the analysis

**Supplementary Table 1**. Clinical and demographic variables comparing individuals included and excluded in the ADVANCE Trial pretreatment drug resistance analytic sample

|  | **Included in Primary Outcome Analysis (n=826)** | **Included in Snapshot Analyses but not Primary Outcome (n=48)** | **Excluded from Analytic Dataset**  **(n=179)** | ***P*-value^a^** |
| --- | --- | --- | --- | --- |
| Female sex (n, %) | 494 (59.8%) | 27 (56.3%) | 102 (57.0%) | <0.001 |
| Age (median, IQR) | 32 (27-38) | 30 (25-33) | 31 (26-37) | 0.04 |
| Married or Partner (n, %) | 168 (20.4%) | 10 (20.8%) | 34 (19.0%) | 0.91 |
| Tertiary education (n, %) | 69 (8.4%) | 8 (16.7%) | 14 (7.8%) | 0.13 |
| Employed (n, %) | 519 (63.8%) | 20 (41.7%) | 102 (58.0%) | 0.005 |
| Pretreatment CD4 count (n, %) |  |  |  | 0.83 |
| $\leq$200 cells/$u$L | 259 (31.4%) | 12 (25.0%) | 51 (28.5%) |  |
| 201-350 cells/$u$L | 247 (30.0%) | 15 (31.3%) | 49 (27.4%) |  |
| 351-500 cells/$u$L | 157 (19.0%) | 11 (22.9%) | 37 (20.7%) |  |
| >500 cells/$u$L | 163 (19.7%) | 10 (20.8%) | 42 (23.5%) |  |
| Pretreatment viral load (n, %) |  |  |  | 0.04 |
| <10,000 copies/mL | 260 (31.5%) | 24 (50.0%) | 61 (34.1%) |  |
| 10,000-100,000 copies/mL | 377 (45.6%) | 20 (41.7%) | 83 (46.4%) |  |
| >100,000 copies/mL | 189 (22.9%) | 4 (8.3%) | 35 (19.6%) |  |
| Study arm (n, %) |  |  |  | 0.45 |
| DTG/TAF/FTC | 272 (32.9%) | 17 (35.4%) | 62 (34.6%) |  |
| DTG/TDF/FTC | 285 (34.5%) | 11 (22.9%) | 55 (30.7%) |  |
| EFV/TDF/FTC | 269 (32.6%) | 20 (41.7%) | 62 (34.6%) |  |
| Presence of WHO-defined pretreatment drug resistance (n, %) | 112 (86.4%) | 10 (20.8%) | 11 (11.5%) | 0.30 |
| Pill count^b,c^ (n, %) |  |  |  | 0.75 |
| <90% | 45 (5.5%) | 3 (10.3%) | 9 (5.1%) |  |
| 90-95% | 81 (9.9%) | 2 (6.9%) | 20 (11.4%) |  |
| >95% | 696 (84.7%) | 24 (82.8%) | 146 (83.4%) |  |

^a^*P*-values represent statistical tests comparing those included and excluded from the analytic dataset, using chi-squared testing for categorical variables and Mann-Whitney non-parametric tests for median age.

^b^Low adherence by self-report was missing over 50% participants in the non-analyzed groups and was not included in this table

^c^Pill count was calculated at each visit by study pharmacists, capped at 100%, then averaged across the 96-week observation period

**Supplementary Table 2**. Multivariable logistic regression models for virologic success in the ADVANCE Trial comparing definitions for virologic success.^a^

|  | **Efavirenz vs Dolutegravir^b^** | | **Presence of WHO-defined pretreatment drug resistance^c^** | |
| --- | --- | --- | --- | --- |
| **Covariable** | **Adjusted Odds Ratio (95%CI)** | ***P*-value^d^** | **Adjusted Odds Ratio (95%CI)** | ***P*-value^d^** |
| Primary Outcome^e^ | 1.02 (0.67, 1.57) | 0.92 | 0.38 (0.23, 0.61) | <0.001 |
| Secondary Outcome^f^ | 1.97 (1.06, 3.66) | 0.03 | 0.25 (0.13, 0.48) | <0.001 |
| 48-week Snapshot^g^ | 2.01 (1.32, 3.05) | 0.001 | 0.41 (0.25, 0.68) | 0.001 |
| 96-week Snapshot^g^ | 1.79 (1.23, 2.61) | 0.002 | 0.35 (0.22, 0.55) | <0.001 |
| Change in log10 viral load from baseline to 12 weeks^h^ | 0.15 (0.07, 0.23) | <0.001 | -0.14 (-0.25, -0.04) | 0.007 |

^a^Models are adjusted for sex, age, marital/partners status, educational attainment, employment, pretreatment CD4 count, pretreatment viral load, self-reported treatment adherence, and pill count adherence during study observation

^b^Estimates indicate the odds ratio estimate from multivariable logistic regression models comparing dolutegravir versus efavirenz as the reference category such that an odds ratio greater than one suggests higher odds of virologic success for individuals in dolutegravir arms

^c^Estimates indicate the odds ratio estimate from multivariable logistic regression models comparing those with versus without WHO-defined pretreatment drug resistance as the reference category such that an odds ratio greater than one suggests higher odds of virologic success for individuals with resistance

^d^*P*-values represent results of two-sided tests of significance for coefficients of the outcome variable in multivariable logistic regression models.

^e^Primary outcome: Virologic success in our primary outcome was defined as achievement of a sustained viral load <1000 copies/mL from 12 weeks, <200 copies/mL from 24 weeks, and <50 copies/mL from 48 weeks onwards. Individuals who are censored after 48-weeks with virologic suppression are considered as achieving virologic success.

^f^Secondary outcome: Virologic success in our secondary outcome was defined as the absence of two consecutive visits with a viral load >200 copies/mL. Individuals who are censored with a single viral load >200 copies/mL are considered failures, whereas those who discontinue with virologic suppression are considered as achieving virologic success.

^g^48 and 96-week Snapshot outcome refer to Food and Drug Administration-defined Snapshot outcomes for HIV therapeutic clinical trials

^h^Mean estimated change in log_10_ viral load was assessed as continuous variable using a linear regression model. Each one-unit change in the outcome corresponds to a log_10_ change in viral load between baseline and week 12.

**Supplementary Table 3.** Virologic success in the ADVANCE Trial by the presence of the K103N mutation prior to treatment initiation.^a^ *P*-values for the total cohort and treatment arms represent results of chi-squared tests. *P*-values for the interaction terms represent two-sided tests of significance for interaction terms between pre-treatment drug resistance and efavirenz vs dolutegravir terms in logistic regression models.

|  | **Total Cohort** | | | **Efavirenz arm** | | | **Dolutegravir arms** | | | **Interaction**  ***P*-value^b^** |
| --- | --- | --- | --- | --- | --- | --- | --- | --- | --- | --- |
|  | **PDR** | **No PDR** | ***P*-value^b^** | **PDR** | **No PDR** | ***P*-value^b^** | **PDR** | **No PDR** | ***P*-value^b^** |  |
| Primary Outcome^c^ | 34/49 (69%) | 611/724 (84%) | 0.006 | 7/8 (88%) | 215/251 (86%) | 0.88 | 27/41 (66%) | 396/473 (84%) | 0.004 | 0.35 |
| Secondary Outcome^d^ | 42/48 (88%) | 654/696 (94%) | 0.08 | 7/8 (88%) | 219/236 (93%) | 0.57 | 35/40 (88%) | 435/460 (95%) | 0.07 | 0.92 |
| 48-week Snapshot^e^ | 38/53 (72%) | 637/763 (84%) | 0.03 | 6/10  (60%) | 213/267  (80%) | 0.13 | 32/43 (74%) | 424/496 (86%) | 0.05 | 0.81 |
| 96-week Snapshot^e^ | 33/53 (63%) | 600/763 (79%) | 0.006 | 5/10 (50%) | 198/267 (74%) | 0.07 | 28/43 (65%) | 402/496 (81%) | 0.01 | 0.82 |
| Mean change in log10 viral load from baseline to 12 weeks (SD) | 2.67 (0.92) | 2.65 (0.80) | 0.88 | 2.14 (0.51) | 2.59 (0.77) | 0.08 | 2.79 (0.95) | 2.68 (0.82) | 0.44 | 0.33 |

PDR: presence of WHO-defined pretreatment drug resistance

^a^Individuals with nucleoside reverse transcriptase inhibitor resistance aside from K103N are excluded from these analyses

^b^*P*-values for the total cohort and treatment arms represent results of chi-squared tests for the primary and secondary outcomes and results of two sided t tests for the change in viral load outcome. *P*-values for the interaction terms represent two-sided tests of significance for interaction terms between pre-treatment drug resistance and efavirenz vs dolutegravir terms in logistic regression models.

^c^Primary outcome: Virologic success in our primary outcome was defined as achievement of a sustained viral load <1000 copies/mL from 12 weeks, <200 copies/mL from 24 weeks, and <50 copies/mL from 48 weeks onwards. Individuals who are censored after 48-weeks with virologic suppression are considered as achieving virologic success.

^d^Secondary outcome: Virologic success in our secondary outcome was defined as the absence of two consecutive visits with a viral load >200 copies/mL. Individuals who are censored with a single viral load >200 copies/mL are considered failures, whereas those who discontinue with virologic suppression are considered as achieving virologic success.

^e^48 and 96-week Snapshot outcome refer to Food and Drug Administration-defined Snapshot outcomes for HIV therapeutic clinical trial

**Supplementary Table 4.** Virologic success in the ADVANCE Trial by the presence of WHO-defined pretreatment minority drug resistance as defined by a mutation frequency 2-20%.^a^

|  | **Total Cohort** | | | **Efavirenz arm** | | | **Dolutegravir arms** | | | **Interaction**  ***P*-value^b^** |
| --- | --- | --- | --- | --- | --- | --- | --- | --- | --- | --- |
|  | **PDR** | **No PDR** | ***P*-value^b^** | **PDR** | **No PDR** | ***P*-value^b^** | **PDR** | **No PDR** | ***P*-value^b^** |  |
| Primary Outcome^c^ | 32/36 (89%) | 575/680 (85%) | 0.48 | 8/10 (80%) | 207/239 (87%) | 0.55 | 24/26 (92%) | 368/441 (83%) | 0.23 | 0.22 |
| Secondary Outcome^d^ | 34/35 (97%) | 614/654 (97%) | 0.43 | 9/10 (90%) | 209/224 (93%) | 0.69 | 25/25 (100%) | 405/430 (94%) | 0.22 | N/A^f^ |
| 48-week Snapshot^e^ | 32/41 (78%) | 600/713 (84%) | 0.30 | 10/10 (100%) | 203/255 (80%) | 0.11 | 22/31 (71%) | 397/458 (87%) | 0.02 | N/A^f^ |
| 96-week Snapshot^e^ | 30/41 (73%) | 565/713 (79%) | 0.35 | 10/10 (100%) | 188/255 (74%) | 0.06 | 20/31 (65%) | 377/458 (82%) | 0.01 | N/A^f^ |
| Mean change in log10 viral load from baseline to 12 weeks (SD) | 2.65  (0.78) | 2.66  (0.79) | 0.96 | 2.41  (0.73) | 2.62  (0.75) | <0.001 | 2.75  (0.78) | 2.68  (0.62) | 0.69 | 0.53 |

PDR: presence of WHO-defined pretreatment drug resistance

^a^Individuals with drug resistance at mutation frequencies >20% are excluded from these analyses

^b^*P*-values for the total cohort and treatment arms represent results of chi-squared tests for the primary and secondary outcomes and results of two sided t tests for the change in viral load outcome. *P*-values for the interaction terms represent two-sided tests of significance for interaction terms between pre-treatment drug resistance and efavirenz vs dolutegravir terms in logistic regression models.

^c^Primary outcome: Virologic success in our primary outcome was defined as achievement of a sustained viral load <1000 copies/mL from 12 weeks, <200 copies/mL from 24 weeks, and <50 copies/mL from 48 weeks onwards. Individuals who are censored after 48-weeks with virologic suppression are considered as achieving virologic success.

^d^Secondary outcome: Virologic success in our secondary outcome was defined as the absence of two consecutive visits with a viral load >200 copies/mL. Individuals who are censored with a single viral load >200 copies/mL are considered failures, whereas those who discontinue with virologic suppression are considered as achieving virologic success.

^e^48 and 96-week Snapshot outcome refer to Food and Drug Administration-defined Snapshot outcomes for HIV therapeutic clinical trial

^f^Unable to calculate Interaction term due to 0 cells

**Supplementary Table 5**. Logistic regression models for 96-week virologic success in the ADVANCE Trial stratified by participants in the efavirenz-based and dolutegravir based arms.^a^

|  | **Dolutegravir Arms** | | **Efavirenz Arms** | |
| --- | --- | --- | --- | --- |
| **Covariable** | **Adjusted Odds Ratio (95%CI)** | ***P*-value** | **Adjusted Odds Ratio (95%CI)** | ***P*-value^b^** |
| Female Sex | 0.78 (0.46, 1.31) | 0.34 | 0.82 (0.37, 1.79) | 0.61 |
| Age (each year) | 1.02 (0.99, 1.06) | 0.23 | 1.02 (0.97, 1.08) | 0.41 |
| Married or Partner | 1.33 (0.66, 2.67) | 0.42 | 0.55 (0.23, 1.32) | 0.18 |
| Tertiary education | 0.66 (0.31, 1.42) | 0.29 | 2.57 (0.47, 14.04) | 0.28 |
| Employed | 2.17 (1.31, 3.57) | 0.002 | 1.04 (0.48, 2.27) | 0.92 |
| Pretreatment CD4 count |  |  |  |  |
| $\leq$200 cells/$u$L | REF |  | REF |  |
| 201-350 cells/$u$L | 1.49 (0.79, 2.80) | 0.22 | 0.91 (0.35, 2.38) | 0.86 |
| 351-500 cells/$u$L | 1.13 (0.54, 2.36) | 0.74 | 0.83 (0.28, 2.49) | 0.74 |
| >500 cells/$u$L | 1.17 (0.56, 2.47) | 0.67 | 0.77 (0.24, 2.45) | 0.66 |
| Pre-treatment viral load |  |  |  |  |
| <10,000 copies/mL | REF |  | REF |  |
| 10,000-100,000 copies/mL | 0.54 (0.29, 1.01) | 0.06 | 0.39 (0.15, 1.05) | 0.06 |
| >100,000 copies/mL | 0.45 (0.21, 0.96) | 0.04 | 0.27 (0.09, 0.81) | 0.02 |
| Low self-reported adherence^c^ (n, %) | 0.38 (0.23, 0.63) | <0.001 | 0.44 (0.21, 0.94) | 0.03 |
| Pill count adherence (n, %)^d^ |  |  |  |  |
| <90% | REF |  | REF |  |
| 90-95% | 1.60 (0.58, 4.44) | 0.37 | 20.04 (2.66, 151.1) | 0.004 |
| 95-100% | 2.65 (1.09, 6.47) | 0.03 | 7.06 (1.76, 28.41) | 0.006 |
| **Presence of WHO-defined pretreatment drug resistance** | **0.45 (0.25, 0.79)** | **0.006** | **0.23 (0.08, 0.68)** | **0.008** |

^a^Virologic success in our primary outcome was defined as achievement of a sustained viral load <1000 copies/mL from 12 weeks, <200 copies/mL from 24 weeks, and <50 copies/mL from 48 weeks onwards. Individuals who are censored after 48-weeks with virologic suppression are considered as achieving virologic success.

^b^*P*-values represent results of two-sided tests of significance for coefficients in multivariable logistic regression models.

^c^Low adherence defined as self-report of less than perfect adherence in the four days prior to any study visits during the observation period

^d^Pill count was calculated at each visit by study pharmacists, capped at 100%, then averaged across the 96-week observation period

**Supplementary Table 6**. Comparison of pre- versus on-treatment WHO-defined reverse transcriptase drug resistance mutation patterns in the entire cohort, and by treatment group

| **NRTI Mutations** |  |  |  |  |  |  |  |  |  |  |  |
| --- | --- | --- | --- | --- | --- | --- | --- | --- | --- | --- | --- |
| **Total Cohort** |  |  |  | **Efavirenz Arm** |  |  |  | **Dolutegravir Arms** |  |  |  |
|  | Pre-treatment Sequencing | |  |  | Pre-treatment Sequencing | |  |  | Pre-treatment Sequencing | |  |
| On Treatment Sequencing | No NRTI Resistance | NRTI Resistance | Total | On Treatment Sequencing | No NRTI Resistance | NRTI Resistance | Total | On Treatment Sequencing | No NRTI Resistance | NRTI Resistance | Total |
| No NRTI Resistance | 27 (82%) | 0 (0%) | 27 | No NRTI Resistance | 11 (69%) | 0 (0%) | 11 | No NRTI Resistance | 16 (94%) | 0 (0%) | 16 |
| NRTI Resistance | 6 (18%) | 5 (100%) | 11 | NRTI Resistance | 5 (31%) | 1 (100%) | 6 | NRTI Resistance | 1 (6%) | 4 (100%) | 5 |
| Total | 33 | 5 | 38 | Total | 16 | 1 | 17 | Total | 17 | 4 | 21 |
|  |  |  |  |  |  |  |  |  |  |  |  |
| **NNRTI Mutations** |  |  |  |  |  |  |  |  |  |  |  |
| **Total Cohort** |  |  |  | **Efavirenz Arm** |  |  |  | **Dolutegravir Arms** |  |  |  |
|  | Pre-treatment Sequencing | |  |  | Pre-treatment Sequencing | |  |  | Pre-treatment Sequencing | |  |
| On Treatment Sequencing | No NNRTI Resistance | NNRTI Resistance | Total | On Treatment Sequencing | No NNRTI Resistance | NNRTI Resistance | Total | On Treatment Sequencing | No NNRTI Resistance | NNRTI Resistance | Total |
| No NNRTI Resistance | 17 (81%) | 2 (12%) | 19 | No NNRTI Resistance | 6 (60%) | 0 (0%) | 6 | No NNRTI Resistance | 11 (100%) | 2 (20%) | 13 |
| NNRTI Resistance | 4 (19%) | 15 (88%) | 19 | NNRTI Resistance | 4 (40%) | 7 (100%) | 11 | NNRTI Resistance | 0 (0%) | 8 (80%) | 8 |
| Total | 21 | 17 | 38 | Total | 10 | 7 | 17 | Total | 11 | 10 | 21 |

**Supplementary Table 7.** Assessment of collinearity between included covariates in our multivariable regression model of our primary outcome

| **Variable** | **Variation Initiation Factor (VIF)** | **1/VIF** |
| --- | --- | --- |
| Female Sex | 1.1 | 0.908709 |
| Age (each year) | 1.14 | 0.877584 |
| Married or Partner | 1.08 | 0.92839 |
| Tertiary education | 1.02 | 0.98108 |
| Employed | 1.11 | 0.903705 |
| Pretreatment CD4 count |  |  |
| $\leq$200 cells/$u$L | REF | -- |
| 201-350 cells/$u$L | 1.45 | 0.690362 |
| 351-500 cells/$u$L | 1.49 | 0.67171 |
| >500 cells/$u$L | 1.62 | 0.618711 |
| Pre-treatment viral load |  |  |
| <10,000 copies/mL | REF | -- |
| 10,000-100,000 copies/mL | 1.49 | 0.671991 |
| >100,000 copies/mL | 1.66 | 0.603334 |
| Low self-reported adherence | 1.1 | 0.911344 |
| Pill count adherence |  |  |
| <90% | REF | -- |
| 90-95% | 2.71 | 0.368918 |
| 95-100% | 2.85 | 0.350324 |
| Efavirenz vs dolutegravir | 1.03 | 0.972675 |
| Presence of WHO-defined pretreatment drug resistance | 1.04 | 0.959658 |

**Supplementary Table 8.** Virologic success in the ADVANCE Trial by the presence of WHO-defined pretreatment drug resistance in the entire analytic dataset and restricted to sequences with an average coverage depth >1,000X. *P*-values for the total cohort and treatment arms represent results of chi-squared tests for the primary and secondary outcomes and results of two sided t tests for the change in viral load outcome.

|  | **Total Cohort** | | | **Restricted to Average Coverage Depth >1,000X** | | |
| --- | --- | --- | --- | --- | --- | --- |
|  | **PDR** | **No PDR** | ***P*-value^a^** | **PDR** | **No PDR** | ***P*-value^a^** |
| Primary Outcome^b^ | 73/112 (65%) | 606/714 (85%) | <0.001 | 67/102 (66%) | 560/659 (85%) | <0.001 |
| Secondary Outcome^c^ | 86/105 (82%) | 646/687 (94%) | <0.001 | 79/96 (82%) | 596/634 (94%) | <0.001 |
| 48-week Snapshot^d^ | 84/122 (69%) | 630/752 (84%) | <0.001 | 76/112 (68%) | 578/692 (84%) | <0.001 |
| 96-week Snapshot^d^ | 71/122 (58%) | 593/752 (79%) | <0.001 | 64/112 (57%) | 546/692 (79%) | <0.001 |
| Mean change in log10 viral load from baseline to 12 weeks (SD) | 2.63  (0.95) | 2.66 (0.79) | 0.78 | 2.58 (0.12) | 2.65 (0.80) | 0.50 |

^a^*P*-values represent results of chi-squared testing for the primary and secondary outcomes and results of two sided t tests for the change in viral load outcome.

^b^Primary outcome: Virologic success in our primary outcome was defined as achievement of a sustained viral load <1000 copies/mL from 12 weeks, <200 copies/mL from 24 weeks, and <50 copies/mL from 48 weeks onwards. Individuals who are censored after 48-weeks with virologic suppression are considered as achieving virologic success.

^c^Secondary outcome: Virologic success in our secondary outcome was defined as the absence of two consecutive visits with a viral load >200 copies/mL. Individuals who are censored with a single viral load >200 copies/mL are considered failures, whereas those who discontinue with virologic suppression are considered as achieving virologic success.

^d^48 and 96-week Snapshot outcome refer to Food and Drug Administration-defined Snapshot outcomes for HIV therapeutic trials

PDR: presence of WHO-defined pretreatment drug resistance

## **Supplementary Figure 1.** Change in log_10_ viral load from the baseline enrolment visit to week 12 by treatment arm and presence or absence of World Health Organization-defined pre-treatment drug adherence mutations to reverse transcriptase. For each box and whisker plots, the central white line represents the median of the distribution, the black box represents the 75% interquartile range, the whiskers represent 1.5 times the interquartile range and dots each represent outliers. *P*-values represent results of two-sided t-tests.

**Supplementary Figure 2**. Kaplan-Meier curves demonstrating time to virologic suppression by treatment arm and presence or absence of World Health Organization-defined pre-treatment drug adherence mutations to reverse transcriptase. P-values represent results of two-sided log-rank testing

**Supplementary Figure 3.** Distribution of read depth coverage among samples included in the analysis
