# Supplementary material for: Reduced efficacy of HIV-1 integrase inhibitors in patients with drug resistance mutations in reverse transcriptase
Source: Nat Commun. 2020 Dec 1;11:5922. doi: 10.1038/s41467-020-19801-x (PMC7708638; doi:10.1038/s41467-020-19801-x)
Supplement: Supplementary file 3 — Reporting Summary [file 41467_2020_19801_MOESM3_ESM.pdf]

nature research

Corresponding author(s): Ravindra Gupta
NCOMMS-20-28625

Last updated by author(s): Sep 22, 2020

# Reporting Summary

Nature Research wishes to improve the reproducibility of the work that we publish. This form provides structure for consistency and transparency in reporting. For further information on Nature Research policies, see our Editorial Policies and the Editorial Policy Checklist.

## Statistics

For all statistical analyses, confirm that the following items are present in the figure legend, table legend, main text, or Methods section.

| n/a | Confirmed | |
|---|---|---|
| ☐ | ☒ | The exact sample size (*n*) for each experimental group/condition, given as a discrete number and unit of measurement |
| ☐ | ☒ | A statement on whether measurements were taken from distinct samples or whether the same sample was measured repeatedly |
| ☐ | ☒ | The statistical test(s) used AND whether they are one- or two-sided<br>*Only common tests should be described solely by name; describe more complex techniques in the Methods section.* |
| ☐ | ☒ | A description of all covariates tested |
| ☐ | ☒ | A description of any assumptions or corrections, such as tests of normality and adjustment for multiple comparisons |
| ☐ | ☒ | A full description of the statistical parameters including central tendency (e.g. means) or other basic estimates (e.g. regression coefficient) AND variation (e.g. standard deviation) or associated estimates of uncertainty (e.g. confidence intervals) |
| ☐ | ☒ | For null hypothesis testing, the test statistic (e.g. $F$, $t$, $r$) with confidence intervals, effect sizes, degrees of freedom and $P$ value noted<br>*Give P values as exact values whenever suitable.* |
| ☒ | ☐ | For Bayesian analysis, information on the choice of priors and Markov chain Monte Carlo settings |
| ☒ | ☐ | For hierarchical and complex designs, identification of the appropriate level for tests and full reporting of outcomes |
| ☐ | ☒ | Estimates of effect sizes (e.g. Cohen's *d*, Pearson's *r*), indicating how they were calculated |

*Our web collection on statistics for biologists contains articles on many of the points above.*

## Software and code

Policy information about availability of computer code

| | |
|---|---|
| Data collection | No software was used for data collection. |
| Data analysis | Data analysis was conducted with Stata version 15.1. All analyses were conducted with pre-packaged Stata code. A copy of the analytic code will be made available to reviewers, editors, or any requesting individual upon request. |

For manuscripts utilizing custom algorithms or software that are central to the research but not yet described in published literature, software must be made available to editors and reviewers. We strongly encourage code deposition in a community repository (e.g. GitHub). See the Nature Research guidelines for submitting code & software for further information.

## Data

Policy information about availability of data

All manuscripts must include a data availability statement. This statement should provide the following information, where applicable:

- Accession codes, unique identifiers, or web links for publicly available datasets
- A list of figures that have associated raw data
- A description of any restrictions on data availability

A dataset will be made available to reviewers, editors, or any requesting individual upon request.

# Field-specific reporting



April 2020

# Life sciences study design

All studies must disclose on these points even when the disclosure is negative.

| | |
|---|---|
| Sample size | The sample size for the ADVANCE clinical trial was determined as follows: "A sample size of 350 patients per group was estimated to provide 80% power to establish noninferior efficacy for the TAF-based regimen as compared with the standard-care regimen and for the TDF-based regimen as compared with the standard-care regi- men.20 The additional comparison of the DTG- containing regimens (TAF-based vs. TDF-based) was also prespecified. These calculations assumed a noninferiority margin of −10 percentage points, as recommended by the FDA,21 with an assumed 80% efficacy in the standard-care group." |
| Data exclusions | Data exclusions were made for study participants with missing or failed genotype testing as described in the results section and displayed in the consort diagram in the study schema Figure 1. |
| Replication | Data analytic code for primary and secondary outcomes was replicated by two separate study investigators (MJS and BS) and compared until identical. All failed HIV-1 RNA viral deep sequencing specimens were repeated. No other replication experiments was conducted |
| Randomization | Treatment allocation in the ADVANCE clinical trial was randomized using centralized, computer generated randomization procedures. |
| Blinding | The ADVANCE clinical trial was an open-label study |

# Reporting for specific materials, systems and methods

We require information from authors about some types of materials, experimental systems and methods used in many studies. Here, indicate whether each material, system or method listed is relevant to your study. If you are not sure if a list item applies to your research, read the appropriate section before selecting a response.

## Materials & experimental systems

| n/a | Involved in the study | |
|---|---|---|
| ☒ | ☐ | Antibodies |
| ☒ | ☐ | Eukaryotic cell lines |
| ☒ | ☐ | Palaeontology and archaeology |
| ☒ | ☐ | Animals and other organisms |
| ☐ | ☒ | Human research participants |
| ☐ | ☒ | Clinical data |
| ☒ | ☐ | Dual use research of concern |

## Methods

| n/a | Involved in the study | |
|---|---|---|
| ☒ | ☐ | ChIP-seq |
| ☒ | ☐ | Flow cytometry |
| ☒ | ☐ | MRI-based neuroimaging |

## Human research participants

Policy information about studies involving human research participants

| | |
|---|---|
| Population characteristics | The study enrolled non-pregnant individuals over 12 years old with HIV infection and naive to antiretroviral therapy from HIV clinics in Johannesburg, South Africa. |
| Recruitment | The ADVANCE trial is an open-label, non-inferiority, phase three clinical trial comparing three regimens for the initial treatment of HIV. Individuals were recruited from 11 public HIV clinics in Johannesburg, South Africa. All study visits and data collection procedures were performed at one of two research clinics in Johannesburg operated by the study staff. Consenting participants were randomized in a 1:1:1 ratio to (i) tenofovir disoproxil fumarate (TDF), emtricitabine (FTC), EFV; (ii) TDF, FTC, DTG, or (iii) tenofovir alafenamide fumarate (TAF), FTC, and DTG. In this sub-analysis, we included only individuals who consented to storage of blood and completed successful sequencing of their pre-treatment HIV-1 RNA. The restriction to this sub-study could contribute selection bias to our study. To account for this, we analyzed differences between participants who did and did not contribute to this sub-study, and conducted all analyses of our outcomes in multivariable adjusted regression models to minimize contributions of potential confounders. |
| Ethics oversight | The study was approved by the institutional review board at the University of the Witwatersrand. All study participants gave written informed consent to participate. |

Note that full information on the approval of the study protocol must also be provided in the manuscript.

## Clinical data

Policy information about clinical studies

All manuscripts should comply with the ICMJEguidelines for publication of clinical research and a completedCONSORT checklist must be included with all submissions.

| | |
|---|---|
| Clinical trial registration | https://clinicaltrials.gov/ct2/show/NCT03122262 |
| Study protocol | Primary results of the ADVANCE clinical trial and the full protocol are available here: https://www.nejm.org/doi/full/10.1056/ |

NEJMoa1902824

| | |
|---|---|
| Data collection | The trial enrolled residents of inner-city Johannesburg from February 2017 through May 2018. Inclusion criteria were an age of 12 years or older, a weight of 40 kg or more, a viral load of 500 copies or more per milliliter, and a creatinine clearance of more than 60 ml per minute (Cockcroft–Gault formula) in patients 19 years of age or older or more than 80 ml per minute (modified Cockcroft–Gault formula) in those younger than 19 years of age. Among the exclusion criteria were more than 30 days of treatment with any form of ART, any ART within the past 6 months, pregnancy, or cur- rent treatment for tuberculosis. |
| Outcomes | For this analysis, our primary outcome of interest was 96-week virologic success, which we defined as sustained a viral load <1000 copies/mL from 12 weeks through 96 weeks, <200 copies/mL from 24 weeks through 96 weeks, and <50 copies/mL from 48 weeks through 96 weeks. Individuals censored with virologic suppression at 48-weeks or after are considered to have achieved virologic success. Individuals who did not complete 12 weeks of observation are not included in this analysis (but are included in the 48 and 96-week Food and Drug Administration [FDA] Snapshot sensitivity outcomes as failures, as described below). We derived this definition to reflect treatment response in individuals who attain and maintain virologic suppression over the course of study observation. We estimated the proportion of participants who achieved virologic suppression by the presence or absence of PDR for the total cohort, and by EFV versus DTG treatment regimens. Finally, to assess the contribution of PDR on virologic response to therapy, we also considered a virologic potency outcome defined by the change in log10 viral load from enrolment to week 12, and assessed for time to first virologic suppression using Kaplan-Meier survival methods. In the survival analyses, individuals were censored at the time of first virologic suppression and considered failures if that occurred with a detectable viral load. |
